# Changes Over a Decade in Anthropometry and Fitness of Elite Austrian Youth Soccer Players

**DOI:** 10.3389/fphys.2019.00333

**Published:** 2019-03-28

**Authors:** Christoph Gonaus, Jürgen Birklbauer, Stefan J. Lindinger, Thomas L. Stöggl, Erich Müller

**Affiliations:** ^1^Department of Sport and Exercise Science, University of Salzburg, Salzburg, Austria; ^2^Department of Food and Nutrition and Sport Science, University of Gothenburg, Gothenburg, Sweden

**Keywords:** football, performance development, testing, talent selection, training control

## Abstract

Increases in physical (e.g., high-intensity running and sprinting), technical (e.g., passing rate), and tactical (e.g., player density) aspects made elite level soccer more challenging within the past years. The aim of the study was to evaluate whether these evolutions are also been reflected in changes in anthropometric and fitness characteristics between former (2002 to 2005) and current (2012 to 2015) elite Austrian youth development center (U13 to U14) and soccer academy (U15 to U18) players. A battery of anthropometric, general and soccer-specific fitness tests was conducted annually at the end of each year. Independent *t*-test and Cohen’s *d* (ES) were calculated to compare the two four-year periods (2530 vs. 2611 players) at each age group separately. Current players were significantly faster in 20 m sprint (ES = 0.26–0.50) and reaction test (ES = 0.15–0.39, except for U18), but less flexible at sit-and-reach (ES = –0.19 to –0.55), in all age categories. Whereas height (ES = 0.26–0.32), body mass (ES = 0.11–0.18) and countermovement jump (ES = 0.24–0.26) increased significantly at youth development center level, current academy players performed superior at shuttle sprint (ES = 0.21–0.59), hurdles agility run (ES = 0.24–0.49), and endurance run (ES = 0.11–0.20). These changes over time in speed, change-of-direction ability, lower-body power, coordination, and endurance were attributed to modern training approaches (e.g., modified games and change-of-direction drills) and modifications in selection politics (e.g., coaches favor speed and decision-making skills).

## Introduction

National soccer associations as well as professional clubs spend an increasing amount of money and time to identify, select and develop young soccer players (Williams and Reilly, 2000). Effective resource management, including early selection and subsequent talent promotion of a few players (Williams and Reilly, 2000; Vaeyens et al., 2008), and economical reward has led to the installation of national development programs and youth soccer academies around the world (Reilly et al., 2000; Darby et al., 2007). Apart from the financial profit, these “centers of excellence” (Reilly et al., 2000) provide a holistic approach (Stratton et al., 2004) aiming to offer their most talented players an optimized environment from technical, tactical, physiological, socio-psychological, and academic perspective to achieve future professional level (Relvas et al., 2010; Mills et al., 2014b).

More recently, periodically fitness testing has gained greater acceptance within the soccer academy setting (Paul and Nassis, 2015). Besides both monitoring performance improvements and comparisons to normative values, coaches are able to effectively adjust their training interventions to the players’ individual strengths and weaknesses (Svensson and Drust, 2005; Lidor et al., 2009). The predictive value of sprint and repeated sprint ability, change-of-direction speed, and endurance to future career progression (Murr et al., 2018) emphasizes the importance of fitness testing in youth soccer. Additionally, significant relationships were detected for certain position-specific match running activities and the performance in sprint, power, and endurance tests (Buchheit et al., 2010; Castagna et al., 2010). With regard to injury prevention screening, the increasing risk of sustaining a hamstring injury due to insufficient hip flexibility (Henderson et al., 2010) further highlights the need for an adequate athletic preparation in youth soccer. Moreover, the academy-to-first-team transition requires an appropriate fitness level to cope with the increased physical training load and playing schedule in adult soccer (Finn and McKenna, 2010).

Within the past years, evolutions of game speed, match structure and play patterns made elite level soccer more technically and physically demanding (Wallace and Norton, 2014). Between the seasons 2006/07 and 2012/13, total distance covered by English Premier League players remained relatively constant (plus 2%) and within the seasonal match-to-match variation of 2.4% (Rampinini et al., 2007). In contrast, high-intensity running (≥19.8 km h^-1^) and sprinting (>25.1 km h^-1^) activities substantially increased both in distance (plus 30 and 35%) and frequency (plus 50 and 85%) over this period (Barnes et al., 2014), even though higher match-to-match variabilities of 17.7 and 30.8% need to be considered for high-intensity running distance and sprint distance (Gregson et al., 2010). Since actions performed at high intensities are decisive within match-winning situations (Faude et al., 2012), one might expect that the upward trend in high-intensity actions during match play has also been accompanied by changes in players’ fitness.

However, the reported evolutions of soccer players’ fitness level in both youth and senior soccer players are inconsistent, even though different nations and levels of play need to be accounted for. For instance, countermovement jump and maximal aerobic power (VO_2max_) remained fairly stable between 1995 and 2010 in Norwegian elite players (Haugen et al., 2013; Tonnessen et al., 2013). In addition, whereas Haugen et al. (2013) demonstrated a small improvement in sprint test performance of the Norwegian sample, Martinez-Santos et al. (2016) even reported small deteriorations in countermovement jump, 5 and 15 m sprint of Spanish third Division players over the last two decades. With respect to youth soccer, Carling et al. (2012) reported a consistency in selection criteria over time, since almost no significant changes in player size, maturity and functional characteristics (10, 20, 40 m sprint, countermovement jump, anaerobic power, VO_2max_, and quadriceps strength) of French youth players on academy entry between 1992 and 2003 were found. On the other hand, Elferink-Gemser et al. (2012) attributed the significant improvements in intermittent endurance capacity between 2000/01 and 2009/10 seasons in under 13 (U13) to under 19 (U19) players to increases in both training hours and quality.

Previous studies on the evolution of youth soccer players’ fitness over time consist of data recorded until 2003 (Carling et al., 2012) or 2010 (Elferink-Gemser et al., 2012) and are limited to evaluations of few academies and/or a small number of athletic characteristics. Based on a unique, longitudinal, and nationwide dataset, the aim of the study was to evaluate whether anthropometric and fitness characteristics have changed between former (2002 to 2005) and current (2012 to 2015) elite Austrian youth soccer players across six age groups (U13 to U18). Given the reported progression in adult match play, it was hypothesized that fitness characteristics, in particular sprint, power and endurance performances, have positively evolved over the investigated decade.

## Materials and Methods

In Austria, since 2001, talented male youth soccer players are promoted systematically into a nationwide development program from the age of 10 (Gonaus and Müller, 2012). Until age group U14, a selected player will receive two to four extra training sessions per week at one of the 29 accredited youth development centers, but still competes for his home club. These youth development centers are funded both by the federal and the national football association. At the age of 14 years, the most talented players are drafted into one of 12 (13 until 2008) youth soccer academies and subsequently compete in a nationwide championship at U15, U16 and U18 level. Academies belong to either a professional club or a federal state federation and are co-funded by the Austrian Football Association [Österreichischer Fußball-Bund (ÖFB)]. Regulations for academy accreditation include infrastructural and economic criteria, cooperation with schools and companies, administrative and technical (full-time coaches) personnel as well as medical, scientific and psychological staff (Öfb, 2017). The cut-off date for the competition categories in Austrian soccer runs from 1st January to 31st December (e.g., U13 players of the 2002/03 season were born in 1990).

Data were collected within a project launched by the ÖFB, the Department of Sport and Exercise Science of the University of Salzburg and the Institute for Sports Medicine and Science (IMSB Austria) with the aim to scientifically guide the progress of the top Austrian youth soccer players between the ages of 12 to 18 years. Since 2001, approximately 8000 male youth development center players and youth soccer academy players have performed a battery of anthropometric, general and soccer-specific fitness tests once, in autumn (U13 to U14), or twice, in summer and winter (U15 to U18), a year. All players and parents signed a training agreement with the ÖFB, who, for their part, gave their permission to the scientific processing of the data. The procedures were in accordance with the ethical standards of the Declaration of Helsinki and were approved by the local university ethics committee. Parts of the dataset have been used in a previous study on anthropometric and fitness predictors of future career progression (Gonaus and Müller, 2012).

### Participants

For the current investigation, 2530 players (tested from 2002 to 2005) and 2611 players tested 10 years later (2012 to 2015) were analyzed. Measurements were taken annually at the end of the year in September or October (U13 to U14) and November, December or January (U15 to U18), respectively. To minimize the effects of one single season, each four consecutive years were summarized per age group and subsequently defined as “former” (2002 to 2005) and “current” (2012 to 2015) period. Due to illness, injuries, ordinary fluctuation of players or any other reasons for non-participation, some players have been tested only once during this four-year interval, whereas others have completed up to four tests in consecutive age groups. Initially, 4058 (2002 to 2005) and 4448 (2012 to 2015) measurements were analyzed. Since some height and body mass data were missing in the years 2002 and 2003, slightly smaller group sizes were available for the anthropometric evaluation. In terms of the fitness characteristics, incomplete datasets, which did not meet the criterion of at least 8 out of 9 tests (U13 to U14) and 9 out of 10 tests (U15 to U18), were excluded. Thus, the following subgroups remained for the age groups and the two four-year periods (2002 to 2005 vs. 2012 to 2015): U13 (*n* = 890 vs. *n* = 967), U14 (999 vs. 906), U15 (769 vs. 869), U16 (550 vs. 742), U17 (474 vs. 483), U18 (261 vs. 283).

### Procedures

Date of birth, height and body mass were recorded in advance of the fitness test battery. Tests were exclusively conducted by experienced researchers and students from either the University of Salzburg or the IMSB Austria. To limit influences of the circadian rhythm and to ensure repeatability, tests were executed on an indoor surface at the same time of day, in the same order, using the same measurement systems (Drust et al., 2005; Svensson and Drust, 2005). The sequence of the tests was in accordance with Harman (2008), who suggested to start with non-fatiguing measurements and tests requiring high-skill movements, to follow with tests for agility and power, and to finish with endurance tests. Subsequent to a standardized 15 min warm-up, the participants started with the first trials of 20 m linear sprint and foot tapping, followed by two attempts of sit-and-reach and 2 kg standing medicine ball throw, respectively. Afterward, they performed the second trials of 20 m sprint and foot tapping. Players were then split into four groups, each passing randomly through one attempt of reaction test, and two trials of hurdles agility run, 5 × 10 m shuttle sprint and jumps (countermovement and drop jump). Finally, only the U15 to U18 players conducted a 20 m multi-stage endurance run. For those tests where two attempts were admitted, only the better score was retained for statistical analyses (Gonaus and Müller, 2012).

### Anthropometric Characteristics

Height (1 cm) was recorded using a portable stadiometer SECA 217 (SECA, Hamburg, Germany). Body mass (measured to the nearest 0.1 kg) was assessed on players in sportswear and without shoes using an electronic flat scale SECA 813 (SECA, Hamburg, Germany). Body mass index (BMI; 0.1 kg m^-2^) was calculated as body mass in kilograms divided by height in meter squared (Nuttall, 2015).

### Fitness Characteristics

The current test battery included a series of general and soccer-specific fitness tests targeting to track elite players’ state of fitness throughout the talent development program and aiming to provide coaches with an extensive picture of their players’ athletic level. The rationale behind measuring speed, agility, and endurance in youth soccer is described in Paul and Nassis (2015). The predictive validity of the test battery was justified by Gonaus and Müller (2012), who reported that future U18 to U21 youth national team players outperform their non-drafted counterparts at an earlier age (14 to 17 years) in almost all physiological measures. For a detailed description of each single test setup and procedure, readers are referred to Gonaus and Müller (2012). With reference to their principal component analysis, the 12 fitness test variables are categorized into three components: “speed” (5, 10, 20 m sprint; 5 × 10 m shuttle sprint), “coordination and endurance” (hurdles agility run, reaction test, foot tapping, and endurance run) and “power and flexibility” (countermovement jump, drop jump, medicine ball throw, and sit-and-reach).

#### Speed

Infrared timing gates (Brower Timing Systems, UT, United States) were used in all sprint tests (0.01 s) with players starting in a standing position 0.5 m behind the first timing gate. Linear sprint speed was measured by 20 m sprint and the corresponding 5 and 10 m split times. A 10 min break interval was given between the two trials. Soccer-specific change-of-direction speed was assessed using the 5 × 10 m shuttle sprint, in which players have to complete five 10 m sprints continuously with each 180° turns. Two attempts were separated by a 5 min break interval.

In a sample of 41 male youth soccer academy players, test-retest (one week apart) intraclass correlation coefficient (ICC) values of 0.78–0.85 were displayed for 10 and 20 m sprint as well as 5 × 10 m shuttle sprint. A low ICC of 0.56 was found in 5 m sprint (Gonaus and Müller, 2013).

#### Coordination and Endurance

The hurdles agility run (0.01 s) was used to quantify general agility. Players had to perform a roll forward; then, always passing the middle pole, they had to jump over the hurdles (located at every side of a cross-shaped running path) and crawl back under them. Hurdles height was adjusted to body size, with a plus of 5 cm in body size corresponding to a 2 cm increase in hurdles height. A 5 min break was given between the two trials. Reaction test was performed to measure multi choice reaction time of the lower limbs in four possible directions: right-front, right-back, left-front and left-back. The mean reaction time (1 ms) of 20 random stimuli was recorded with the computer based system of Fitronic (Fitronic Inc., Bratislava, Slovakia). Maximum speed of lower limb movement was assessed via foot tapping on a force plate applying piezoelectric sensors (Kistler Instrument Corporation, Winterthur, Switzerland). Single foot contacts over 5 s were multiplied by 2 and divided by 5 to obtain the bipedal cycles per second (0.1 Hz). Two attempts were separated by a 10 min break. Aerobic endurance was measured by a 20 m multi-stage endurance run with the speed given by an audio signal. The protocol included repetitive 20 m runs for 3 min at 7.92, 9.72, 11.52 and 13.32 km h^-1^, interspersed by a 90 s break to determine blood lactate concentrations (Laktat Analyser Biosen 5040, EKF Industrie-Elektronik, Barleben, Germany). The speed corresponding to 4 mmol l^-1^ (0.1 km h^-1^) was determined from the lactate-velocity-curve using an exponential fit.

Whereas the ICC of 0.48 was low for the reaction test, hurdles agility run, foot tapping and endurance run displayed moderate to high values (ICC = 0.79–0.93) (Gonaus and Müller, 2013).

#### Power and Flexibility

Countermovement jump and drop jump were used to assess lower-body power and explosive strength. Each two attempts with arm swing separated by a 15 s break were performed without shoes on the Kistler force plate (Kistler Instrument Corporation, Winterthur, Switzerland). Countermovement jump height (0.1 cm) was calculated via ground reaction force measurement. For the drop jump, U15 to U18 players were instructed to perform a maximal two-legged vertical jump as quickly as possible after dropping down from a 40 cm platform (30 cm for U13 to U14 players). Jump high (0.1 cm), measured via flight time, and ground contact time (1 ms) were recorded and subsequently applied into the formula for the drop jump coefficient (to the nearest 0.01) described in Gonaus and Müller (2012). Upper-limb power was determined performing the 2 kg standing overhead medicine ball throw (0.1 m). With a maximum of one-step run-up, two attempts were administered immediately consecutive. Two successive trials of sit-and-reach (1 cm) were conducted to assess general flexibility.

Whereas countermovement jump and drop jump showed acceptable ICC values of 0.74–0.75, high values (ICC = 0.94–0.97) were found for 2 kg medicine ball throw and sit-and-reach (Gonaus and Müller, 2013).

#### Statistical Analyses

Age group specific means and standard deviations were calculated for each four-year period. Microsoft Excel (Version 2010, Microsoft, Seattle, WA, United States) was used for graphics and descriptive calculations. Inferential statistics were computed using IBM SPSS statistics version 24.0 (SPSS Inc., Chicago, IL, United States). A threshold for outlier detection was set separately for each age category and period, rejecting all data displaying *z*-scores < –4.0 and > 4.0 (Hopkins et al., 2009). *T*-tests for independent samples were used to identify differences in the anthropometric and fitness characteristics between the former and current periods for each age group separately. The significance level was set at *p* < 0.05. Effect sizes (Cohen’s *d*; ES) were calculated according to the formula for the independent *t*-test described in Sink and Stroh (2006) with the recommendation of 0.2, 0.5 and 0.8 for small, medium and large effects, respectively (Cohen, 1992). For a deeper understanding of the mechanisms behind the hypothesized changes in players’ fitness over the years, relative birth quartile distributions were compared between former and current players using Chi-squared tests and the corresponding Cramer’s *V* over all age categories, as well as for each age group separately.

## Results

**FIGURE 1:**
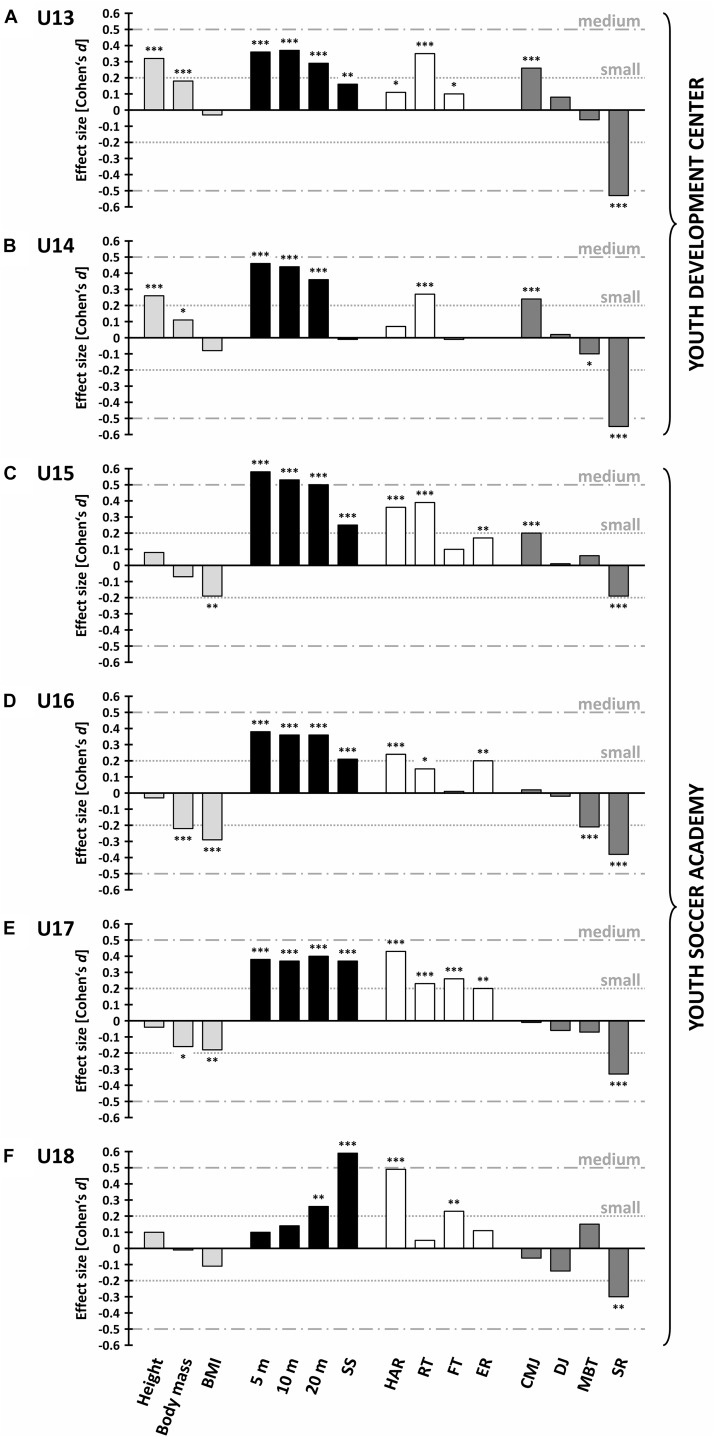
Differences (Cohen’s *d*) in “anthropometric” (light gray), “speed” (black), “coordination and endurance” (white) and “power and flexibility” (dark gray) characteristics between former (2002 to 2005) and current (2012 to 2015) U13 **(A)**, U14 **(B)**, U15 **(C)**, U16 **(D)**, U17 **(E)** and U18 **(F)** soccer players. BMI = body mass index, 5/10/20 m = 5/10/20 m sprint, SS = 5 × 10 m shuttle sprint, HAR = hurdles agility run, RT = reaction test, FT = foot tapping, ER = 20 m multi-stage endurance run, CMJ = countermovement jump, DJ = drop jump, MBT = 2 kg overhead medicine ball throw, SR = sit-and-reach. ^∗^ *p* < 0.05; ^∗∗^ *p* < 0.01; ^∗∗∗^ *p* < 0.001. Positive Cohen’s *d* values indicate superior performance of current players, whereas negative Cohen’s *d* values denote performance decreases.

Figure 1 illustrates the increases (positive Cohen’s *d*), decreases (negative Cohen’s *d*) and statistical significances in anthropometry and performance of “speed,” “coordination and endurance,” and “power and flexibility” between former and current U13 to U18 soccer players.

### Anthropometry

Current youth development center players were significantly taller (2.2–2.4 cm, *p* < 0.001) than former players, with small effect sizes of 0.32 (U13) and 0.26 (U14). Significant, but only trivial effects of 0.11–0.18 (0.9–1.2 kg, *p* < 0.03) were found for body mass and no significant changes (*p* > 0.10) were detected for BMI. At academy level, while no systematic changes over time were displayed for height (*p* > 0.12), current players were significantly lighter in U16 (–1.7 kg, ES = –0.22, *p* < 0.001) and U17 (–1.1 kg, ES = –0.16, *p* = 0.02), resulting in a decrease of BMI (–0.3 to –0.5 kg m^-2^, ES = –0.18 to –0.29, *p* < 0.01; except for U18) compared to former players (Table 1).

**Table 1:** Descriptive, *M* ±*SD* (*n*), and inferential analyses for the anthropometric variables.

	2002 to 2005	2012 to 2015	Mean difference [95% CI]	*t*-statistic	*p*	Cohen’s *d* [95% CI]
Body height (cm)				
U13	151.4 ± 7.2 (684)	153.8 ± 7.6 (979)	2.4 [1.6; 3.1]	6.37	< 0.001	0.32 [0.22; 0.42]
U14	158.3 ± 8.6 (724)	160.5 ± 8.5 (929)	2.2 [1.4; 3.0]	5.21	< 0.001	0.26 [0.16; 0.36]
U15	169.6 ± 8.2 (590)	170.2 ± 7.9 (906)	0.7 [–0.2; 1.5]	1.54	0.123	0.08 [–0.02; 0.19]
U16	175.0 ± 6.4 (446)	174.8 ± 6.6 (774)	–0.2 [–1.0; 0.5]	–0.57	0.566	–0.03 [–0.15; 0.08]
U17	177.6 ± 5.9 (381)	177.4 ± 6.2 (521)	–0.2 [–1.0; 0.6]	–0.52	0.606	–0.03 [–0.17; 0.10]
U18	178.4 ± 5.7 (217)	179.1 ± 6.1 (319)	0.6 [–0.4; 1.6]	1.16	0.245	0.10 [–0.07; 0.28]
Body mass (kg)				
U13	41.1 ± 6.5 (684)	42.3 ± 6.9 (979)	1.2 [0.6; 1.9]	3.61	< 0.001	0.18 [0.08; 0.28]
U14	47.0 ± 8.3 (724)	47.9 ± 8.2 (929)	0.9 [0.1; 1.7]	2.20	0.028	0.11 [0.01; 0.21]
U15	58.6 ± 9.6 (590)	58.0 ± 8.8 (906)	–0.6 [–1.6; 0.3]	–1.32	0.186	–0.07 [–0.17; 0.03]
U16	65.8 ± 7.9 (446)	64.0 ± 7.9 (774)	–1.7 [–2.6; -0.8]	–3.69	< 0.001	–0.22 [–0.34; -0.10]
U17	69.4 ± 7.2 (381)	68.3 ± 7.0 (521)	–1.1 [–2.1; -0.2]	–2.33	0.020	–0.16 [–0.29; -0.03]
U18	71.4 ± 6.7 (217)	71.3 ± 6.7 (319)	–0.1 [–1.3; 1.1]	–0.16	0.871	–0.01 [–0.19; 0.16]
Body mass index (kg m^-2^)				
U13	17.8 ± 1.7 (684)	17.8 ± 1.7 (979)	0.0 [–0.2; 0.1]	–0.53	0.593	–0.03 [–0.12; 0.07]
U14	18.6 ± 1.9 (724)	18.5 ± 1.8 (929)	–0.1 [–0.3; 0.0]	–1.62	0.105	–0.08 [–0.18; 0.02]
U15	20.3 ± 2.0 (590)	19.9 ± 1.8 (906)	–0.4 [–0.6; -0.2]	–3.49	0.001	–0.19 [–0.29; -0.08]
U16	21.4 ± 1.7 (446)	20.9 ± 1.8 (774)	–0.5 [–0.7; -0.3]	–4.89	< 0.001	–0.29 [–0.41; -0.17]
U17	22.0 ± 1.6 (381)	21.7 ± 1.7 (521)	–0.3 [–0.5; -0.1]	–2.64	0.009	–0.18 [–0.31; -0.05]
U18	22.4 ± 1.6 (217)	22.2 ± 1.6 (319)	–0.2 [–0.4; 0.1]	–1.23	0.218	–0.11 [–0.28; 0.06]


### Speed

Linear sprint times significantly improved over the decade for all age groups (0.02–0.07 s, ES = 0.26–0.58, *p* < 0.001), with the exception of 5 and 10 m sprint for U18 (*p* > 0.09). Especially at U15 level, medium effect sizes ranging from 0.50 to 0.58 (0.04–0.07 s, *p* < 0.001) were found. Regarding the 5 × 10 m shuttle sprint, a significant performance increase (0.08 s, ES = 0.16, *p* = 0.001) was displayed for U13 players, but no significant change (*p* = 0.87) over the decade was reported for U14 players. At academy level, current players showed significant superior shuttle sprint performances (0.09–0.23 s, ES = 0.21–0.59, *p* < 0.001), with the largest effect size of 0.59 (0.23 s, *p* < 0.001) detected for U18 (Table 2).

**Table 2:** Descriptive, *M* ± *SD* (*n*), and inferential analyses for the factor “speed.”

	2002 to 2005	2012 to 2015	Mean difference [95% CI]	*t*-statistic	*p*	Cohen’s *d* [95% CI]
5 m sprint (s)				
U13	1.18 ± 0.07 (890)	1.15 ± 0.05 (963)	0.02 [0.02; 0.03]	7.75	< 0.001	0.36 [0.27; 0.45]
U14	1.15 ± 0.07 (998)	1.12 ± 0.06 (906)	0.03 [0.02; 0.04]	10.10	< 0.001	0.46 [0.37; 0.55]
U15	1.10 ± 0.07 (768)	1.07 ± 0.06 (869)	0.04 [0.03; 0.04]	11.52	< 0.001	0.58 [0.47; 0.67]
U16	1.07 ± 0.07 (550)	1.04 ± 0.05 (742)	0.02 [0.02; 0.03]	6.47	< 0.001	0.38 [0.25; 0.48]
U17	1.05 ± 0.07 (474)	1.03 ± 0.05 (483)	0.02 [0.02; 0.03]	5.92	< 0.001	0.38 [0.25; 0.51]
U18	1.04 ± 0.06 (261)	1.03 ± 0.05 (283)	0.01 [0.00; 0.02]	1.16	0.247	0.10 [–0.07; 0.27]
10 m sprint (s)				
U13	2.02 ± 0.09 (890)	1.99 ± 0.08 (963)	0.03 [0.02; 0.04]	7.97	< 0.001	0.37 [0.28; 0.46]
U14	1.97 ± 0.09 (998)	1.93 ± 0.08 (906)	0.04 [0.03; 0.05]	9.66	< 0.001	0.44 [0.35; 0.53]
U15	1.88 ± 0.09 (768)	1.83 ± 0.08 (869)	0.04 [0.04; 0.05]	10.65	< 0.001	0.53 [0.43; 0.63]
U16	1.82 ± 0.08 (550)	1.79 ± 0.07 (742)	0.03 [0.02; 0.04]	6.27	< 0.001	0.36 [0.24; 0.46]
U17	1.79 ± 0.07 (474)	1.77 ± 0.06 (483)	0.03 [0.02; 0.03]	5.75	< 0.001	0.37 [0.24; 0.50]
U18	1.77 ± 0.07 (261)	1.76 ± 0.07 (283)	0.01 [0.00; 0.02]	1.66	0.097	0.14 [–0.03; 0.31]
20 m sprint (s)				
U13	3.52 ± 0.15 (890)	3.48 ± 0.14 (963)	0.04 [0.03; 0.06]	6.33	< 0.001	0.29 [0.20; 0.39]
U14	3.42 ± 0.15 (998)	3.37 ± 0.15 (906)	0.05 [0.04; 0.07]	7.80	< 0.001	0.36 [0.27; 0.45]
U15	3.25 ± 0.15 (768)	3.17 ± 0.13 (869)	0.07 [0.06; 0.09]	10.08	< 0.001	0.50 [0.40; 0.60]
U16	3.13 ± 0.12 (550)	3.08 ± 0.11 (742)	0.04 [0.03; 0.06]	6.41	< 0.001	0.36 [0.25; 0.47]
U17	3.07 ± 0.11 (474)	3.03 ± 0.10 (483)	0.04 [0.03; 0.05]	6.19	< 0.001	0.40 [0.27; 0.53]
U18	3.04 ± 0.11 (261)	3.01 ± 0.10 (283)	0.03 [0.01; 0.04]	3.03	0.003	0.26 [0.09; 0.43]
5 × 10 m shuttle sprint (s)				
U13	12.74 ± 0.53 (888)	12.66 ± 0.52 (965)	0.08 [0.03; 0.13]	3.36	0.001	0.16 [0.07; 0.25]
U14	12.35 ± 0.52 (996)	12.35 ± 0.48 (904)	0.00 [–0.05; 0.04]	–0.16	0.869	–0.01 [–0.10; 0.08]
U15	11.79 ± 0.50 (764)	11.67 ± 0.43 (866)	0.12 [0.07; 0.16]	5.08	< 0.001	0.25 [0.15; 0.35]
U16	11.44 ± 0.44 (550)	11.35 ± 0.39 (741)	0.09 [0.04; 0.13]	3.70	< 0.001	0.21 [0.10; 0.32]
U17	11.29 ± 0.40 (471)	11.14 ± 0.38 (482)	0.14 [0.09; 0.19]	5.71	< 0.001	0.37 [0.24; 0.50]
U18	11.25 ± 0.39 (261)	11.02 ± 0.38 (283)	0.23 [0.16; 0.29]	6.92	< 0.001	0.59 [0.42; 0.77]


### Coordination and Endurance

Only trivial effects of decade were found for youth development center players in hurdles agility run (ES = 0.07–0.11, *p* = 0.02–0.12) and foot tapping (ES = –0.01–0.10, *p* = 0.04–0.80), whereas significant small enhancements (24–33 ms, ES = 0.27–0.35, *p* < 0.001) were displayed for the reaction test. At academy level, current players performed significantly better than former players across all age categories at the hurdles agility run (0.15–0.29 s, ES = 0.24–0.49, *p* < 0.001) and the reaction test (9–28 ms, ES = 0.15–0.39, *p* < 0.02), except for the reaction test at U18 (*p* = 0.54). Foot tapping significantly improved over the decade at U17 and U18 (0.3 Hz, ES = 0.23–0.26, *p* < 0.01). Significant improvements (0.13–0.15 km h^-1^, ES = 0.17–0.20, *p* < 0.01) were obtained for the endurance run over the years, with the exception of U18 (*p* = 0.22) (Table 3).

**Table 3:** Descriptive, *M* ±*SD* (*n*), and inferential analyses for the factor “coordination and endurance.”

	2002 to 2005	2012 to 2015	Mean difference [95% CI]	*t*-statistic	*p*	Cohen’s *d* [95% CI]
Hurdles agility run (s)			
U13	12.99 ± 0.83 (885)	12.89 ± 0.90 (963)	0.10 [0.02; 0.18]	2.40	0.017	0.11 [0.02; 0.20]
U14	12.55 ± 0.80 (990)	12.49 ± 0.87 (904)	0.06 [–0.02; 0.14]	1.57	0.118	0.07 [–0.02; 0.16]
U15	12.04 ± 0.73 (761)	11.79 ± 0.67 (865)	0.25 [0.18; 0.32]	7.26	< 0.001	0.36 [0.26; 0.46]
U16	11.56 ± 0.62 (542)	11.41 ± 0.61 (733)	0.15 [0.08; 0.22]	4.31	< 0.001	0.24 [0.13; 0.36]
U17	11.40 ± 0.59 (468)	11.14 ± 0.62 (470)	0.26 [0.18; 0.34]	6.60	< 0.001	0.43 [0.30; 0.56]
U18	11.29 ± 0.61 (258)	11.00 ± 0.56 (273)	0.29 [0.19; 0.39]	5.69	< 0.001	0.49 [0.32; 0.67]
Reaction test (ms)			
U13	757 ± 101 (850)	724 ± 88 (965)	33 [24; 42]	7.35	< 0.001	0.35 [0.25; 0.44]
U14	693 ± 94 (956)	668 ± 86 (905)	24 [16; 33]	5.83	< 0.001	0.27 [0.18; 0.36]
U15	631 ± 83 (727)	604 ± 59 (868)	28 [20; 35]	7.48	< 0.001	0.39 [0.28; 0.48]
U16	582 ± 69 (532)	573 ± 53 (737)	9 [2; 16]	2.45	0.014	0.15 [0.03; 0.25]
U17	572 ± 70 (459)	557 ± 52 (483)	14 [6; 22]	3.55	< 0.001	0.23 [0.10; 0.36]
U18	557 ± 60 (255)	554 ± 51 (283)	3 [–6; 12]	0.62	0.539	0.05 [–0.12; 0.22]
Foot tapping (Hz)			
U13	11.0 ± 1.0 (890)	11.1 ± 1.1 (964)	0.1 [0.0; 0.2]	2.10	0.036	0.10 [0.01; 0.19]
U14	11.5 ± 1.1 (997)	11.5 ± 1.1 (905)	0.0 [–0.1; 0.1]	–0.25	0.804	–0.01 [–0.10; 0.08]
U15	12.5 ± 1.2 (769)	12.6 ± 1.1 (869)	0.1 [0.0; 0.2]	1.96	0.050	0.10 [0.00; 0.19]
U16	13.2 ± 1.1 (548)	13.2 ± 1.1 (742)	0.0 [–0.1; 0.1]	0.09	0.930	0.00 [–0.11; 0.12]
U17	13.4 ± 1.2 (474)	13.7 ± 1.1 (483)	0.3 [0.1; 0.4]	3.96	< 0.001	0.26 [0.13; 0.38]
U18	13.6 ± 1.1 (261)	13.9 ± 1.1 (283)	0.3 [0.1; 0.4]	2.67	0.008	0.23 [0.06; 0.40]
20 m multi-stage endurance run (km h^-1^)			
U15	11.79 ± 0.84 (649)	11.92 ± 0.62 (845)	0.13 [0.05; 0.20]	3.18	0.002	0.17 [0.06; 0.27]
U16	11.94 ± 0.84 (534)	12.09 ± 0.62 (732)	0.15 [0.06; 0.23]	3.39	0.001	0.20 [0.08; 0.30]
U17	12.06 ± 0.82 (463)	12.20 ± 0.60 (468)	0.14 [0.05; 0.23]	2.99	0.003	0.20 [0.07; 0.32]
U18	12.18 ± 0.71 (252)	12.25 ± 0.62 (273)	0.07 [–0.04; 0.19]	1.23	0.218	0.11 [–0.06; 0.28]


### Power and Flexibility

Current players showed significant better countermovement jump performances (1.0–1.2 cm, ES = 0.20–0.26, *p* < 0.001) at the age groups U13 to U15, but these small effects became trivial (*p* > 0.45) at older age groups. Only trivial effects of decade were found for drop jump (ES = –0.14–0.08, *p* > 0.10) and 2 kg medicine ball throw (ES = –0.10–0.15, *p* > 0.02) across all age groups, with the exception of the medicine ball throw at U16 (–0.3 m, ES = –0.21, *p* < 0.001), where the former players significantly outperformed the current ones. Concerning the sit-and-reach, former players displayed significant greater flexibility across all age groups (–1.2 to –3.0 cm, *p* < 0.01) with small to medium effect sizes (ES = –0.19 to –0.55) (Table 4).

**Table 4:** Descriptive, *M* ±*SD* (*n*), and inferential analyses for the factor “power and flexibility.”

	2002 to 2005	2012 to 2015	Mean difference [95% CI]	*t*-statistic	*p*	Cohen’s *d* [95% CI]
Countermovement jump (cm)					
U13	26.5 ± 5.1 (890)	27.7 ± 4.2 (967)	1.2 [0.8; 1.6]	5.50	< 0.001	0.26 [0.16; 0.35]
U14	28.8 ± 5.4 (999)	29.9 ± 4.6 (905)	1.2 [0.7; 1.6]	5.18	< 0.001	0.24 [0.15; 0.33]
U15	33.0 ± 5.7 (769)	34.1 ± 4.7 (869)	1.0 [0.5; 1.6]	4.01	< 0.001	0.20 [0.10; 0.30]
U16	36.5 ± 5.3 (550)	36.6 ± 4.8 (742)	0.1 [–0.4; 0.7]	0.42	0.676	0.02 [–0.09; 0.13]
U17	38.1 ± 5.5 (474)	38.0 ± 4.6 (483)	0.0 [–0.7; 0.6]	–0.15	0.882	–0.01 [–0.14; 0.12]
U18	39.6 ± 5.3 (260)	39.3 ± 4.7 (283)	–0.3 [–1.2; 0.5]	–0.75	0.454	–0.06 [–0.23; 0.10]
Drop jump (coeff.)					
U13	5.13 ± 1.74 (886)	5.27 ± 1.84 (965)	0.14 [–0.03; 0.30]	1.63	0.103	0.08 [–0.02; 0.17]
U14	5.69 ± 1.86 (997)	5.71 ± 1.99 (906)	0.03 [–0.15; 0.20]	0.32	0.747	0.01 [–0.08; 0.10]
U15	6.46 ± 1.99 (765)	6.48 ± 1.80 (862)	0.02 [–0.17; 0.20]	0.17	0.861	0.01 [–0.09; 0.11]
U16	7.38 ± 2.07 (544)	7.34 ± 2.01 (738)	–0.04 [–0.26; 0.19]	–0.32	0.751	–0.02 [–0.13; 0.09]
U17	8.09 ± 2.16 (470)	7.96 ± 2.05 (481)	–0.14 [–0.40; 0.13]	–0.99	0.321	–0.06 [–0.19; 0.06]
U18	8.55 ± 2.31 (259)	8.25 ± 2.06 (281)	–0.30 [–0.67; 0.07]	–1.59	0.112	–0.14 [–0.31; 0.03]
2 kg overhead medicine ball throw (m)					
U13	5.9 ± 1.2 (886)	5.8 ± 1.0 (964)	–0.1 [–0.2; 0.0]	–1.20	0.231	–0.06 [–0.15; 0.04]
U14	6.9 ± 1.5 (994)	6.7 ± 1.2 (902)	–0.1 [–0.3; 0.0]	–2.28	0.023	–0.10 [–0.19; -0.01]
U15	8.5 ± 1.5 (765)	8.6 ± 1.4 (867)	0.1 [–0.1; 0.2]	1.15	0.250	0.06 [–0.04; 0.15]
U16	10.0 ± 1.4 (549)	9.7 ± 1.4 (738)	–0.3 [–0.4; -0.1]	–3.69	< 0.001	–0.21 [–0.32; -0.10]
U17	10.7 ± 1.4 (473)	10.6 ± 1.3 (483)	–0.1 [–0.3; 0.1]	–1.00	0.315	–0.06 [–0.19; 0.06]
U18	11.1 ± 1.4 (259)	11.3 ± 1.4 (282)	0.2 [0.0; 0.4]	1.69	0.092	0.15 [–0.02; 0.31]
Sit-and-reach (cm)					
U13	6.2 ± 5.2 (887)	3.4 ± 5.5 (965)	–2.8 [–3.3; -2.3]	–11.29	< 0.001	–0.52 [–0.62; -0.43]
U14	7.6 ± 5.5 (998)	4.6 ± 5.3 (906)	–3.0 [–3.5; -2.5]	–11.98	< 0.001	–0.55 [–0.64; -0.46]
U15	9.7 ± 6.1 (769)	8.6 ± 6.0 (866)	–1.2 [–1.7; -0.6]	–3.84	< 0.001	–0.19 [–0.29; -0.09]
U16	12.5 ± 6.0 (549)	10.2 ± 6.4 (738)	–2.3 [–3.0; -1.7]	–6.71	< 0.001	–0.38 [–0.49; -0.27]
U17	13.0 ± 6.1 (473)	10.9 ± 6.3 (481)	–2.0 [–2.8; -1.3]	–5.12	< 0.001	–0.33 [–0.46; -0.20]
U18	13.0 ± 5.9 (261)	11.1 ± 6.3 (281)	–1.8 [–2.9; -0.8]	–3.49	0.001	–0.30 [–0.47; -0.13]


### Relative Birth Distribution

**FIGURE 2:**
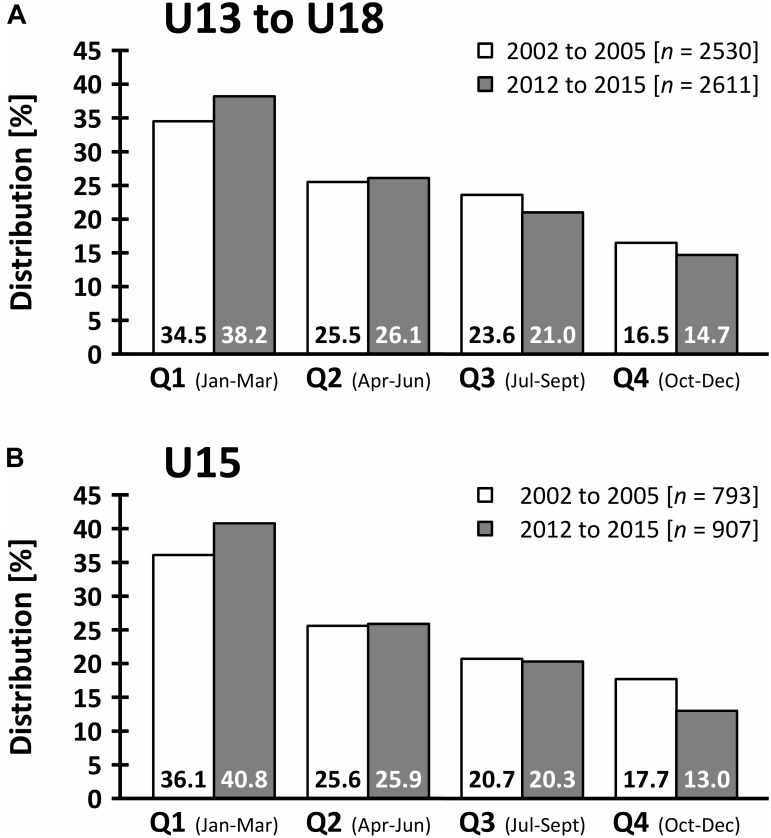
Relative age quarter distribution of former (2002 to 2005) and current (2012 to 2015) players over all age categories **(A)** and in U15 **(B)**.

The comparisons of the birth quartile distributions between former and current players are presented in Table 5. Significant differences, indicating a more pronounced relative age effect in current players, were found over all age categories (Cramer’s *V* = 0.05, *p* = 0.009), and at U15 level (Cramer’s *V* = 0.07, *p* = 0.037; Figure 2).

**Table 5:** Statistical differences of relative birth quartile distributions in former (2002 to 2005) and current (2012 to 2015) youth soccer players.

Category	Period	*n*	Q1 [%]	Q2 [%]	Q3 [%]	Q4 [%]	**X**^2^	*p*	Cramer’s *V*
Total	2002 to 2005	2530	34.5	25.5	23.6	16.5	11.49	0.009	0.05
	2012 to 2015	2611	38.2	26.1	21.0	14.7			
U13	2002 to 2005	905	35.7	29.3	22.3	12.7	2.49	0.477	0.04
	2012 to 2015	982	37.4	26.2	22.6	13.8			
U14	2002 to 2005	1015	32.6	26.5	23.4	17.4	5.42	0.144	0.05
	2012 to 2015	932	37.6	25.3	21.4	15.8			
U15	2002 to 2005	793	36.1	25.6	20.7	17.7	8.51	0.037	0.07
	2012 to 2015	907	40.8	25.9	20.3	13.0			
U16	2002 to 2005	573	40.1	25.7	20.4	13.8	0.07	0.996	0.01
	2012 to 2015	777	39.5	26.1	20.6	13.8			
U17	2002 to 2005	502	37.8	22.5	24.5	15.1	4.02	0.260	0.06
	2012 to 2015	523	40.2	26.0	21.2	12.6			
U18	2002 to 2005	270	34.1	22.6	26.7	16.7	3.87	0.276	0.08
	2012 to 2015	327	37.6	26.3	20.5	15.6			


*Q = relative age quarter; Q1 = January–March, Q2 = April–June, Q3 = July–September, Q4 = October–December.*

## Discussion

The main objective of the study was to evaluate whether anthropometric and fitness characteristics of elite Austrian youth soccer players have changed over one decade. Whereas height and body mass both increased over the investigated period at youth development center level, body mass and BMI decreased at almost all academy-level age groups. Regarding the fitness characteristics, current youth development center players showed superior performances most notably in linear sprint speed, multi choice reaction time and lower-body power. At academy level, the largest improvements over the decade were found in speed, change-of-direction ability, coordination and endurance parameters.

Elite Austrian youth soccer players have become faster, but less flexible, over the last few years, independent of age category. The enhancements in the linear sprint test and multi choice reaction task fit well into the trends of elite adult match play over time, demonstrating improvements in maximal running speed (Barnes et al., 2014) and number of explosive sprints for all playing positions (Bush et al., 2015). The increasing demands in physical (e.g., high-intensity running and sprinting distance), technical (e.g., passing rate), and tactical (e.g., player density) activities (Barnes et al., 2014; Wallace and Norton, 2014), along with the importance of straight line sprinting in game-winning situations (Faude et al., 2012) require players with pronounced speed and decision-making skills. With respect to the latter, multi choice response time has been shown to evoke decision-making processes such as cognitive flexibility and inhibitory control (Stuss et al., 2005), which, for their part, were found to discriminate between playing level in youth soccer (Huijgen et al., 2015). Whereas Austrian youth coaches apparently appreciated speed and decision-making skills over the last few years, probably little attention was put on players’ flexibility. Current players showed a decreased general flexibility across all six age groups. To counteract this negative trend, training contents might potentially be reconsidered in order to prevent muscle strain injuries (Witvrouw et al., 2003), in particular hamstring strain injuries (Wan et al., 2017).

In contrast to Carling et al. (2012), who reported a consistency in selection philosophy on academy entry between 1992 and 2003, current players on youth development center entry were taller (1.6%), heavier (2.9%), and performed superior in linear sprint (up to 1.7%), reaction time (4.4%), and countermovement jump (4.5%) compared to former players. At the subsequent academy selection at U15, current players outperform the former players at linear speed (up to 3.6%), change-of-direction ability (1.0%), general agility (2.1%), reaction time (4.4%), endurance (1.1%), and lower-body power (3.0%). These improvements in speed, coordination, endurance, and power characteristics over time may be caused by training induced changes and/or variations in selection philosophy. Regarding the latter, both U13 and U15 selections are decisive stages in the promotion of Austrian youth soccer players, because admission to such programs should ensure superior physical development (Wrigley et al., 2014), high quality coaching (Mills et al., 2014a) and thus, increases the likelihood of reaching future professional level (Meylan et al., 2010). Given the advantages of chronological age and early maturation on anthropometric, speed, agility, endurance, and power characteristics (Malina et al., 2004; Figueiredo et al., 2010; Gil et al., 2014), the improvements of current players may be at least partially ascribed to an increased selection bias favoring early born and/or more mature players (Meylan et al., 2010) nowadays. Based on the notably changes in anthropometric values at youth development center selection, advanced biological maturation of current U13 players might be a reason. Although speculative, this assumption could be supported by the findings of Müller et al. (2017), showing that only 2.3% of Austrian youth development center players were late maturing these days, compared to 23.9% early maturers. In addition, since youth players born close to the cut-off date benefit from an advantage in soccer experience of up to one year (Helsen et al., 2005) and thus, are more likely to be treated as “talented” (Furley and Memmert, 2016), subsequent analyses were performed to compare birth quartile distributions between former and current players. In accordance with a previous study examining the changes in relative age effect over time in professional soccer (Helsen et al., 2012), no decreases of this phenomenon were found in elite Austrian youth soccer over the last decade. Despite considerable research over the last years (Musch and Grondin, 2001; Cobley et al., 2009), a significant more pronounced relative age effect in current players was detected over all age categories, and at U15 level. The more distinct relative age effect at U15 level may be attributed to the high competition to gain an academy place (Musch and Grondin, 2001) and could have amplified the enhancements in almost all fitness variables over the decade at this particular age group.

Besides changes in selection politics, fitness improvements may be ascribed to both enhancements in training hours and training quality over the last few years. A reduced BMI (0.9–2.3%) and superior performances in favor of current players were found in general agility (1.3–2.6%) and soccer-specific change-of-direction speed (0.8–2.0%) as well as in endurance (0.6–1.3%) during the academy years. As height remained stable over time at U15 to U18 players, decreases in BMI could be primarily associated with the reductions in body mass. More specifically, since BMI and body fat percentage highly correlated in 12 to 14-year old soccer players (Nikolaidis, 2012), lower BMI values might be due to reductions in fat mass, resulting in leaner players nowadays. Within this context, both decreased BMI and reduced body fat percentage were related to superior endurance performance (Wong et al., 2009; Nikolaidis, 2012) and a better final league ranking (Lago-Penas et al., 2011) in youth soccer players. Referring to Elferink-Gemser et al. (2012), the lower BMI and the improvements in endurance performance may be at least partially ascribed to increases in training hours. Admittedly, the amount of training hours was not measured in the present study; however, subsequent to the revision of a youth soccer development program, Tears et al. (2018) showed an increase in training exposure per player over time in a comparable, homogeneous group of academy players. Furthermore, the use of advanced soccer-specific training methods has been recommended to enhance the quality of training (Reilly, 2005). To optimize training time by simultaneously improving physical, technical and tactical requirements for soccer (Clemente et al., 2014), modified games (e.g., small sided games) have recently become very popular in youth soccer (Cronin et al., 2017). In addition, traditional (straight line) sprint training has been extended by more sport-specific change-of-direction speed drills (Sheppard and Young, 2006). Emphasizing on both small sided games and change-of-direction training nowadays might have boosted the improvements in general agility and soccer-specific change-of-direction speed over time in the current sample (Chaouachi et al., 2014).

Even though the present study was based on a unique dataset of the top youth soccer players of an entire country across several years, some limitations need to be considered. The mechanisms behind the fitness progressions over the decade remain unclear, since some athletic improvements could simply be reduced to changes in anthropometric values or birth dates, rather than to training induced evolutions or selection modifications. In addition, the effects of repeated testing (i.e., some players were tested up to four times in consecutive age groups) might be seen as a limitation. Though, similar to Elferink-Gemser et al. (2012), age groups were compared with each other separately. Nevertheless, it is important to highlight that repeated measurements, advanced maturity and relative age could have affected some of the athletic progressions over the years. Further investigations should also document the playing positions since the requirements during match play differ by position, especially between goalkeepers and outfield players (Di Salvo et al., 2008). But we assume that the number of goalkeepers has been almost equal between the two periods. Moreover, reporting training content and the amount of training hours might have been valuable to draw conclusions about changes in training quantity and quality over time.

## Conclusion

Several anthropometric and fitness characteristics improved over time in elite Austrian youth soccer players. Although partially small in magnitude, the progressions in an already preselected, highly talented group of players offer some beneficial information on the former and current fitness standards of young soccer players, and thus, highlight the need for a periodic update of reference values in youth soccer. Analyzing the athletic development trends may assist scientists, coaches and scouts in planning appropriate training interventions, and supports them with selection decisions (Elferink-Gemser et al., 2012). In terms of the latter, the outcomes of the current analyses of birth quartile distributions emphasize the necessity to work on practicable solutions to overcome the relative age disadvantages within the selection process. In addition, coaches and scouts need to be aware that the athletic trends vary by age, as youth development center players evolved mainly in speed, multi choice reaction time and lower-body power, whereas academy players showed the largest progressions in speed, coordination and endurance parameters. Moreover, the evolution of players’ fitness characteristics over a 10-year period highlights the importance of an adequate athletic preparation in order to keep up with the ongoing progression of the physical and physiological game demands. On a final note, apart from training to improve the on-field performance, preventive strategies to avoid injuries need to be integrated into daily workout routines.

## Author Contributions

CG analyzed the data, prepared tables and figures, and wrote the manuscript. All authors conceived and designed the study, contributed to interpretation and manuscript revision, and read and approved the submitted version.

## Conflict of Interest Statement

The authors declare that the research was conducted in the absence of any commercial or financial relationships that could be construed as a potential conflict of interest.
